# Plasma total antioxidant capacity and peroxidation biomarkers in psoriasis

**DOI:** 10.1186/s12929-016-0268-x

**Published:** 2016-07-04

**Authors:** Ilaria Peluso, Arturo Cavaliere, Maura Palmery

**Affiliations:** Center of Nutrition, Council for Agricultural Research and Economics (CREA-NUT), Via Ardeatina 546, Rome, 00178 Italy; Dermopathic Institute of the Immaculate IDI-IRCCS, Via dei Monti di Creta, 104, Rome, 00167 Italy; Department of Physiology and Pharmacology “V. Erspamer”, “Sapienza” University of Rome, P.le Aldo Moro 5, Rome, 00185 Italy

**Keywords:** Human studies, Peroxidation biomarkers, Psoriasis, Total antioxidant capacity

## Abstract

Systemic biomarkers of oxidative stress can be relevant for assessment of psoriasis severity, for prediction of the outcome of therapy and of the development of comorbidities. In this review we aimed to evaluate the relationship between plasma total antioxidant capacity (TAC) and peroxidation biomarkers, as well as their association with dyslipidemia and systemic inflammation in psoriasis. The review of 59 case–control comparisons (from 41 studies) and 17 interventions (from 13 studies) suggests that peroxidation markers are more sensitive than TAC in the evaluation of oxidative stress in psoriasis. Although few studies investigated the effect of treatment on oxidative stress, it seems that biological drugs could be the better choice in the treatment of psoriasis. However, considering the limitations of TAC and plasma peroxidation markers, this review suggests that new methods should be developed in order to evaluate systemic oxidative stress in psoriasis.

## Background

Psoriasis is a common, chronic inflammatory and immune-mediated skin disease [1, 2].

In psoriatic patients, biomarkers could be relevant for distinction between the different clinical variants of the disease, for the assessment of disease activity and severity and for the prediction of the outcome of a therapeutic intervention [1, 2]. In recent years, the great importance of the use of biomarkers for the prediction of the development of comorbidities such as arthritis, cardiovascular diseases (CVD) and metabolic syndrome has been acknowledged [3–7]. In particular, soluble biomarkers have the potential to be useful for screening patients with psoriasis for underlying psoriatic arthritis [2]. Chiu and Ritchlin [5] proposed a model to explain how psoriatic arthritis originates from a cutaneous plaque. The initial inflammatory events originate in the plaque (activation of monocytoid dendritic cells, macrophages and T cells) and lead to an increase of tumor necrosis factor alpha (TNF-α) production. Activated T cells and monocytoid dendritic cells circulate to lymph nodes, joints, and bone marrow [5]. TNF-α overproduction is highlighted in each compartment, but other inflammatory cytokines, such as interleukin (IL)-12, IL-17, IL-22, and IL-23 are also critically important [1, 5]. In particular, a linear relationship has been suggeted between proximal inducers (IL-23 and IL-12) and the T-helper (Th) cell activation [1]. IL-23 activates Th, which subsequently produce IL-17 and IL-22, whereas IL-12 induces the Th1 response [1].

Both Th1 and Th17 cytokines induce an increased generation of reactive oxygen species (ROS) [8], which is involved in the pathogenesis of psoriasis [9]. In fact, increased ROS production originates not only from exogenous agents, such as cigarette smoking [10], but also from endogenous sources, such as the inflammatory responses of leucocytes involving NADPH-oxidase (NOX), inducible nitric oxide synthase (iNOS) and myeloperoxidase (MPO) activation during oxidative burst [8]. It is known that leukocyte-mediated oxidation of the (LDL) contributes to the pathogenesis of atherosclerosis [8]. Oxidized low density lipoproteins (oxLDL) have been suggested to be markers of accelerated atherosclerosis in rheumatoid arthritis and psoriatic arthritis, whereas vitamin A, vitamin E and β-carotene seem to be associated only to the presence of the autoimmune disorders [11]. In a case–control study on patients with psoriasis and sex- and age-matched healthy volunteers, psoriatic skins were shown positive oxLDL staining, whereas there was no staining in non-lesional skin samples from the same subject [12]. Furthermore, the mean levels of cholesterol (CHOL) and triglycerides (TG) in patients with psoriasis were found to be significantly higher than those of healthy subjects [12].

In this context, although psoriasis is traditionally considered a skin-specific inflammatory disease with the exception of coexisting psoriatic arthritis, it has been recognised as a systemic disease and dyslipidemia is one of the comorbidities in psoriatic patients [13–15]. Therefore, the relationship of systemic biomarkers of oxidative stress with lipid profile and inflammatory markers in psoriasis is an interesting topic. The description of the biomarkers of lipid, protein and DNA damage, as well as of antioxidant defences has been recently reviewed in the context of systemic lupus erythematosus [16]. Although isoprostanes are the highly sensitive and specific markers of oxidative stress in patients with psoriasis [17], many studies reported measurements of other markers of peroxidation, such as oxLDL, malondialdehyde (MDA), thiobarbituric acid reactive substance (TBARS), peroxides, dienes and total oxidant capacity (TOC), also named total oxidant status (TOS), as well as the plasma total antioxidant capacity (TAC), also named total antioxidant status (TAS), total antioxidant response (TAR), antioxidant potential (AOP) or non-enzymatic antioxidant capacity (NEAC) [18–62]. While isoprostanes correlated with other markers of lipid peroxidation (e.g., TBARS) [17], in a review of human intervention studies the isoprostanes levels were affected neither by treatments (e.g. green tea, green tea extracts, and epigallocatechin gallate), nor by study design (e.g. bolus or repeated administration), or when NEAC increased after treatment [63]. On the other hand, the majority of the interventions with green tea and its supplements pointed out an increase of NEAC (69 %, *n* = 22/32) [63]. Therefore, lipid peroxidation resulted unrelated to TAC, probably because the latter often measures the antioxidant capacity in an hydrophilic environment [63]. In this review we aimed to evaluate the relationship between these biomarkers, as well as their association with dyslipidemia and systemic inflammation in psoriasis.

## Review

### Case–control studies

A review of forty-one case–control studies [18–58] (Table 1) has been performed. The number of subjects of the studies ranged from 13 [33] to 516 [41]; some studies reported separately data for mild, moderate and severe psoriasis [22, 31, 32, 36, 42, 45], for active and inactive psoriasis [46–49], for smokers and non smokers [20, 30] or for subjects with and without metabolic syndrome [55]. Therefore fifty-nine case–control comparisons were available from forty-one studies (Table 1).

**Table 1:** Case–control studies reporting plasma or serum peroxidation biomarkers and Total Antioxidant Capacity in psoriasis: association with inflammation and lipid profile

Ref.	Case/control	Peroxidation	TAC	Inflammation	Lipid profile
[18]	100/100	↑ MDA			↑ CHOL, LDL
					↔ HDL, TG
[19]	100/100	↑ MDA		↑VAP-1	↑ CHOL, LDL
					↔ HDL, TG
[20]	25/20: 10/10 smokers	↑ MDA			
	15/10 non-smokers	↑ MDA			
[21]	29/18	↑MDA/TBARS	↓	↑ROS	
[22]	30/10: 10 mild		↔		
	10 moderate		↔		
	10 severe		↔		
[23]	35/25	↑ MDA	↓		
[24]	32/32	↑ MDA			
[25]	8/8	↑ peroxides	↓		
[26]	34/37	↑ TBARS, oxLDL	↔	↑ CRP	↑ CHOL, LDL, TG
					↓ HDL
[27]	73/38	↑ TBARS	↔	↑CRP	
[28]	67/35	↑ TBARS			
[29]	58/45	↑ MDA	↓		
[30]	54/62: 28/16 smokers	↑ TOS	↓		↔ CHOL, LDL, HDL, TG
	26/46 non-smokers	↑ TOS	↓		↔ CHOL, LDL, HDL, TG
[31]	23/25: 12 mild (1)	↑ peroxides and dienes		↔ CRP	↔ CHOL, LDL, HDL, TG
	11 severe (2)	↑ peroxides and dienes		↔ CRP	↔ CHOL, LDL, HDL, TG
[32]	55/20: 11 mild,	↑ MDA	↓		
	20 moderate	↑ MDA	↓		
	24 severe	↑ MDA	↓		
[33]	7/6		↑		
[34]	16/16	↑ TBARS	↓		
[35]	59/40	↑ MDA		↑ IL-6	
[36]	90/30: 30 mild	↑ MDA	↓		
	30 moderate	↑ MDA	↓		
	30 severe	↑ MDA	↓		
[37]	39/25	↑ TOC	↓		
[38]	60/47	↑ peroxides	↓		
[39]	32/20	↔ TBARS			
[40]	40/20	↑ peroxides			
[41]	127/389	↑ peroxides			
[42]	100/100: 29 Mild	↑ MDA			↑ CHOL, LDL
					↔ HDL, TG
	60 Moderate	↑ MDA			↑ CHOL, LDL
					↔ HDL, TG
	11 Severe	↑ MDA			↑ CHOL, LDL
					↔ HDL, TG
[43]					↑ CHOL, TG
	30/33	↑ oxLDL		↑ ESR	↔ LDL
					↓ HDL
[44]	24/26	↑ MDA		↑ IL-8	
[45]	90/90: 30 mild	↑ MDA			
	30 moderate	↑ MDA			
	30 severe	↑ MDA			
[46]	40/40: 34 remission	↔ MDA			
	6 acute	↑ MDA			
[47]	60/30: 30 inactive psoriasis	↑ TBARS	↓		↔CHOL, LDL, TG
					↓ HDL
	30 active psoriasis	↑ TBARS	↓		↑ CHOL, LDL, TG ↓ HDL
[48]	60/40: 30 inactive psoriasis	↑ TBARS	↔	↑ CRP	
	30 active psoriasis	↑ TBARS	↓	↑ CRP	
[49]	48/40: 24 inactive psoriasis	↑ TBARS	↔		↔CHOL, LDL, TG
					↓ HDL
	24 active psoriasis	↑ TBARS	↓		↑ CHOL, LDL, TG ↓ HDL
[50]	33/36		↔		
[51]	23/23	↑ MDA			
[52]	45/45	↑ MDA			↑ CHOL, LDL, ↔ HDL, TG
[53]	40/47	↑ TOS	↓		
[54]	30/23	↔ MDA	↔		↔ CHOL, LDL, HDL, TG
[55]	52/25: 25 with MS	↔ TOS	↔		↑ CHOL, LDL, TG
					↓ HDL
	27 without MS	↔ TOS	↔		↔ CHOL, LDL, HDL, TG
[56]	35/35	↑ MDA, oxLDL	↓	CRP, ESR↑	↑ CHOL, LDL, TG ↓ HDL
[57]	67/35	↑ TBARS			
[58]	22/22	↔ MDA			

CHOL: cholesterol; CRP: C-reactive protein; ESR: erythrocyte sedimentation rate; HDL: high-density lipoprotein; IL: interleukin; LDL: low-density lipoprotein; MDA: malondialdehyde; MS: metabolic syndrome; oxLDL: oxidized low-density lipoprotein; ROS: reactive oxygen species; TAC: total antioxidant capacity; TBARS: thiobarbituric acid reactive substance; TG: triglycerides; TOC: total oxidant capacity, also named total oxidant status (TOS); VAP-1: vascular adhesion protein-1; ↑: increased; ↓: decreased; ↔: unchanged

These studies reported measurements of TAC (*n* = 33), of markers of peroxidation (*n* = 54) and some of these measured both markers (28) (Table 1). The majority of the case–control comparisons pointed out an increase of markers of peroxidation (88.9 %, 48/54), whereas decreased level of TAC were reported in 63.6 % of the cases (21/33) (Table 1). Furthermore, the first study that evaluated TAC in psoriasis reported that TAC in patients with psoriasis was significantly higher compared to healthy controls [33]. Although, the percentage of case–control comparisons reporting consistent results between peroxidation markers and TAC was 85.7 % (24/28), increased levels of peroxidation markers were accompanied by increased markers of inflammation in 83.3 % (10/12) of the cases, whereas decreased TAC was associated with increased inflammation only in 50 % (3/6) of the cases (Table 1).

In fact, although some studies found that severity wise decrease in TAC levels [32, 36], Basavaraj et al. [22] reported that there was no significant difference in the average level of TAC of control, mild, moderate and severe groups, despite the increase in serum 8-hydroxy-2’-deoxyguanosine (8-OHdG; a DNA damage biomarker).

On the contrary, clinical severity of psoriasis, determined according to the Psoriasis Area Severity Index (PASI) score, was significantly correlated with the concentrations of MDA [20, 32, 51]. Furthermore, the serum levels of peroxidation markers (MDA, peroxide and dienes) were observed to be significantly increased from mild to moderate and from moderate to severe psoriasis [20, 31, 32, 36, 46] and correlated positively with the duration of the disease [20]. Also levels of MDA in lesional tissues were significantly higher than those in non-lesional tissues [51]. On the contrary, Toker et al. [54] did not find a significant correlation between the percentage of psoriatic lesions and MDA. On the other hand, significant differences were found in TBARS levels between patient with active and inactive psoriasis [47–49]. However it has been also reported that there was no statistically significant difference in serum TBARS between psoriatic patients and healthy volunteers, as well as no statistically significant correlation between disease duration, disease severity and serum levels of TBARS [39]. Relhan et al. [46] comparing two groups of patients, i.e. those with a PASI score of less than 6 and more than 6, did not find significant differences in the two groups in plasma MDA during the acute phase. However, plasma MDA levels were significantly higher in the group of patients with PASI scores more than 6 in the remission phase [46]. These patients achieved remission, however, their mean PASI score as well as MDA levels were still higher than those of the other group of patients [46], suggesting a persistent condition of systemic oxidative stress. It was recently reported [35] that MDA values in psoriatic subjects were similar to those of patients with coronary artery disease (*n* = 59) and a significant positive correlation was found between MDA and the vascular adhesion protein-1 (VAP-1), involved in the migration process of lymphocytes into sites of inflammation [19]. In this context, it is known that multiple molecular events, involving ROS generation and inflammation, are involved in smoking-induced CVD [10]. In this context, it has been suggested that some constituents of cigarette smoke (i.e. nicotine, methyl vinyl ketone, and α,β-unsaturated aldehydes, such as, acrolein and crotonaldehyde) could be responsible for NOX-mediated oxidative stress [10]. NOX has been established as an important source of ROS contributing to the pathogenesis of CVD [10] and it is well known that the respiratory burst of leukocytes was abnormally high in patients with psoriasis [64, 65]. In fact, increased NOX activity and intracellular production of ROS were observed in subjects with psoriasis in conjunction with decreased TAC and increased TBARS [21].

Attawa and Swelam [20] reported a significant increase in serum MDA and in PASI in smokers compared with non-smokers, that increased with increasing the pack-years of smoking. Furthermore, a highly significant difference in the levels of MDA and PASI score was detected among ex-smoker patients in comparison with the smoker patients [20]. On the contrary, Emre et al. [30] reported that, despite significantly higher PASI scores in smoker patients than in non-smoker patients, TAC and TOS levels were similar between smoker and non-smoker patients and both smoker and non-smoker patients had significantly increased TOS levels and decreased TAC levels than healthy subjects. Furthermore, in this study there were no significant correlations between PASI scores and TAC, TOS, TG and CHOL levels in patients with psoriasis [30].

Twenty out of fifty-eight case–control comparisons described also data of lipid profile (Table 1). Only six studies reported increased levels of TG and decreased HDL (Table 1) [26, 43, 47, 49, 55, 56], two of which found high levels of TG only in patients with active psoriasis [47, 49] and another one only in subjects with metabolic syndrome (MS) [55]. On the contrary, high levels of CHOL and LDL were found in 60.0 % of cases (12/20) and in 55 % of cases (11/20), respectively (Table 1). The high levels of CHOL were accompanied by an increase in the markers of peroxidation in 91.7 % (11/12) of cases and by a decrease in TAC only in 60 % (3/5).

Overall case–control studies suggest that markers of peroxidation are more sensitive indexes of oxidative stress than TAC, and are more often associated with inflammation and dyslipidemia.

### Intervention studies

Topical therapy (i.e. corticosteroids, vitamin D3 and its analogues, calcineurin inhibitors, retinoids, dithranol, tar and keratolytic agents such as salicylic acid and urea) is the first line therapy of psoriasis [66]. Phototherapy, including ultraviolet (UV) B and photochemotherapy (psoralen oral or topical with local ultraviolet A, PUVA) are established treatments for psoriasis that are used for those patients in whom topical therapy has failed [66]. On the other hand, ciclosporin and methotrexate (MTX) are the most commonly used systemic therapies to treat psoriasis and will be referred to as systemic non-biological therapies [66]. Biological therapies have been introduced into the management of psoriasis. Three TNF-α antagonists (adalimumab, etanercept and infliximab), and the IL12/23 monoclonal antibody (ustekinumab) are licensed for use in moderate and severe psoriasis [66]. All four agents are approved for use by National Institute for Health and Clinical Excellence in people who have failed to respond to systemic nonbiological therapies including ciclosporin, methotrexate and PUVA or in subjects intolerant to, or having a contraindication to these treatments [66].

We performed a review of seventeen interventions, from thirteen studies (Table 2), regarding topical therapy [26–28, 33, 57], phototherapy [26, 27, 39, 59], drug treatment [29, 60, 61] and biological treatments [21, 44, 62].

**Table 2:** Intervention studies reporting plasma or serum peroxidation biomarkers and Total Antioxidant Capacity in psoriasis: association with inflammation and lipid profile

Ref.	N° subjects: Treatment	Peroxidation	TAC	Inflammation	Lipid profile
	Duration (study design)				
[33]	7: topical treatment^a^		↑		
	Single application				
[28]	67: topical treatment^b^	↓ TBARS			
	20 days				
[57]	67: topical treatment^b^	↓ TBARS			
	20 days				
[26]	10: topical treatment^c^	↓ TBARS	↔	↔ CRP	↔ CHOL, LDL, HDL, TG
		↔ oxLDL			
	11: NB UVB	↔ TBARS, oxLDL	↔	↓ CRP	↔ CHOL, LDL, HDL, TG
	13: PUVA	↔ TBARS, oxLDL	↓	↓ CRP	↔ CHOL, LDL, HDL, TG
	3, 6, and 12 weeks (3 treatments in parallel)				
[27]	10: topical treatment^c^	↓ TBARS	↔	↔ CRP	
	17: NB UVB	↓ TBARS	↔	↓ CRP	
	20: PUVA	↓ TBARS	↔	↓ CRP	
	3, 6, and 12 weeks (2 treatments in parallel)				
[39]	32: BB UVB	↑ TBARS			
	21 weeks (longitudinal)				
[59]	24: NB UVB	↑ TOS	↔	↔ CRP	
	30 sessions (longitudinal)				
[60]	3: MMF	↔ MDA		↔ CRP	
	1 months (longitudinal)				
[29]	58: MTX	↑ MDA	↔		
	6 and 12 weeks (longitudinal)				
[61]	26: MTX	↔ TOS	↔		↓ CHOL, LDL, HDL
	8 weeks (longitudinal)				
[21]	13: Infliximab	↓ MDA	↔	↓ ROS	
	6 months (parallel: treated versus 16 untreated patients)				
[62]	23: Etanercept	↓ peroxides	↑	↓ CRP	↔ CHOL, LDL, HDL, TG
	24 weeks (longitudinal)				
[44]	24: Efalizumab	↓ MDA		↔ IL-8	
	12-week (longitudinal)			↓ Ex-vivo TNF-α and IFN-γ	

BB UVB: broad-band UVB; CHOL: cholesterol; CRP: C-reactive protein; HDL: high-density lipoprotein; IFN: interferon; IL: interleukin; LDL: low-density lipoprotein; MDA: malondialdehyde; MMF: mycophenolate mofetil; MTX: Methotrexate; NB UVB: narrow-band UVB; oxLDL: oxidized low-density lipoprotein; PUVA: photochemotherapy; ROS: reactive oxygen species; TAC: total antioxidant capacity; TBARS: thiobarbituric acid reactive substance; TG: triglycerides; TNF: tumor necrosis factor; TOS: total oxidant status; Topical treatment: ^a^fluocinolone acetonide, ^b^salicyl ointment, cignoline, tar, followed by bethametasone dipropionide with salicyl acid, ^c^calcipotriene (calcipotriol) or betamethasone dipropionate, or a combination of the two; ↑: increased; ↓: decreased; ↔: unchanged

Gavan et al. [33] monitored the level of TAC in 7 males with psoriasis before and after a single application of fluocinolone acetonide 0.025 % ointment to 90 % of the body. The results showed that the plasma level of TAC was significantly increased at 24 h after glucocorticosteroid application. Two other studies [26, 27] did not find changes in TAC after long term treatment with calcipotriene (calcipotriol) or betamethasone dipropionate, or a combination of the two. Moreover, the authors found a significant reduction in TBARS, but not in oxLDL and in C reactive protein (CRP) (Table 2).

Systemic and local immune suppression are involved in the efficacy of phototherapy regimens such as broad-band UVB (BB UVB), narrow-band UVB (NB UVB) and PUVA [67]. Although these therapies reduced CRP in the majority of the interventions (80 %, 4/5) (Table 2), both UV radiation and psoralens generate free radicals, potentially inducing oxidative stress [67]. In the reviewed studies were observed decreased (2/6) [27], unchanged (2/6) [26] and increased (2/6) [39, 59] levels of markers of peroxidation after phototherapy (Table 2). The TAC decreased only in one of the five interventions (i.e. PUVA) (Table 2).

Conflicting results came also from treatment with drugs such as mycophenolate mofetil (MMF, 1 month, begun in a dosage of 1 g/d and increased over 1 wk to 1.5 to 2.0 g/d administered in two divided doses) [60] and MTX (7.5 mg per week for 12 weeks [29] or 80 and 160 mg cumulative dosage for 8 weeks [61]) (Table 2). One study reported that plasma MDA was significantly increased after MTX treatment of psoriasis patients, whereas TAC was not significantly changed after 12 weeks of treatment [29]. Another study [61] reported that there was no statistically significant alteration in serum levels of TAC and TOS, after 8 weeks of MTX therapy. The only study that evaluated the effect of MMF [60] was done only in three patients with psoriasis who had also grade I essential hypertension (140 to 159/90 to 99) and reported mild improvement of psoriasis in two cases and symptoms unchanged in the other.

Among currently available biological drugs for treating psoriasis, TAC or markers of peroxidation were measured after treatment with anti-TNF-α biologics (infliximab via intravenous administration of 5 mg/kg every 8 weeks for 6 months [21] or etanercept subcutaneously at a dosage of 50 mg biweekly for 12 weeks, followed by 25 mg biweekly for a further 12 weeks [62]) and anti-CD11a (efalizumab, weekly 1 mg/kg of body weight subcutaneous injections for 12 weeks [44]). All biologics decreased markers of peroxidation (100 % 3/3) and etanercept increased TAC (Table 2). Decreases in ROS and CRP were reported after infliximab [21] and etanercept [62], respectively.

Although the higher plasma IL-8 levels, compared to healthy sunjects, were unchanged after efalizumab treatment, the latter abrogates TNF-α and IFN-γ *ex–vivo*-induced expression during the mixed leukocyte reaction [44]. Therefore, in all studies the decrease in peroxidation was accompanied by the decrease of at least one marker of inflammation.

As observed in case–control studies lipid peroxidation markers seem to be more sensitive to redox status in psoriasis.

Concerning lipid profile, only MTX treatment decreased CHOL, but this decrease was accompanied by an increase of the values of alanine aminotransferase (ALT), suggesting toxic effects of MTX on the liver [61]. On the other hand, lipid profile did not change after 24 weeks of treatment with etanercept, despite the improvement of redox status, and the decrease of CRP [62] (Table 2).

Although overall the reviewed intervention studies (Table 2) suggest that biological drugs could be the better choice in the treatment of psoriasis, they do not improve dyslipidemia. In this context, Saraceno et al. [68] did not find significant changes in plasma CHOL, LDL, HDL and TG in patients with psoriasis after 48 weeks of treatment with anti-TNF-α (infliximab, etanercept and adalimumab) and efalizumab, whereas a significant increase in the mean body mass index was observed. From that, probably the use of anti-TNF-α should be associated to an energy-restricted diet in order to improve psoriasis symptoms and lipid profile [69] and to prevent the weight gain [70].

## Discussion

**Fig. 1:**
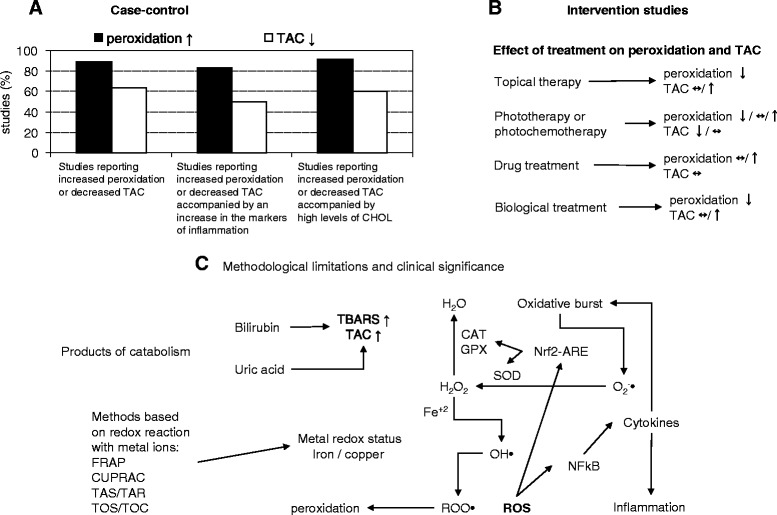
Case–control (**a**) and intervention studies (**b**) suggested that marker of peroxidation could be a more sensitive index of oxidative stress than total antioxidant capacity (TAC). However, some methodological limitations and the relationship between cellular and plasma oxidative stress must be taken into account to properly evaluate the clinical significance of TAC and peroxidation biomarkers in psoriasis (**c**). TAC methods are strongly influenced by the presence of products of catabolism, such as uric acid and bilirubin, the latter is able to react with thiobarbituric acid (TBARS). The clinical significance of methods based on redox reactions with metal ions must consider the potential detrimental effect of reduced metals in conditions of low levels of antioxidant enzymes (**c**). The latter, as well as the inflammatory cytokines are regulated at transcriptional levels by cellular redox status. ↑: increased, ↓: decreased/:or ↔: unchanged; ARE: antioxidant responsive elements; CAT: catalase; CHOL: cholesterol; CUPRAC: cupric reducing antioxidant capacity, Fe: iron; FRAP: ferric reducing antioxidant potential; GPX: glutathione peroxidise; H_2_O_2_: hydrogen peroxide; NF-kB: nuclear factor- kappa B; Nfr2: nuclear factor-erythroid 2-related factor 2; O^2^-•: superoxide radical; OH•: hydroxyl radical; ROO•: peroxyl radicals; ROS: reactive oxygen species; SOD: superoxide dismutase; TOC: total oxidant capacity; TOS: total oxidant status

Both case–control and intervention studies suggested that marker of peroxidation could be a more sensitive index of oxidative stress than TAC. As previously observed and suggested, lipid peroxidation resulted unrelated to TAC, probably because the latter often measures the antioxidant capacity in hydrophilic environment [63]. This could account to the fact that markers of peroxidation are more often associated with dyslipidemia (Fig. 1), but does not explain the association with the inflammatory markers (Fig. 1). However, limitations of the reviwed studies must be taken into account in the interpretation of the results. Furthermore, some methodological consideration should be made and the relationship between cellular and plasma oxidative stress must be taken into account for properly evaluating the clinical significance of TAC and peroxidation biomarkers in psoriasis.

### Limitations of the reviwed studies

A possible explanation of the conflicting data of the reviwed studies could be found in the potential limitations of the these studies.

First of all, the number of patients in individual trials ranged from 3 to 127 (Table 1 and Table 2). The small number of subjects analized in many studies could be below the sample size needed to give an information of both normal range in healthy subjects [25, 33, 34] and oxidative stress in psoriasis before and after treatments [21, 25–27, 60].

Secondly, the included trials varied in terms of study population. The duration of psoriasis sometimes ranged between few months (1–2) and many years (more than 20). In particular Baz et al. [23] reported a duration of the disease ranging between 1 month and 30 years and Coimbra et al. [27] between 2 months and 55 years. Furthermore, PASI score was very variable. For example Attwa et al. [20] reported PASI of 13.8 ± 5.9 in non-smokers and of 34.36 ± 11.8 in smokers, whereas Emre et al. [30] reported PASI of 11.09 ± 4.99 in non-smokers and of 16.35 ± 10.94 in smokers.

Also, when patients were stratified for disease activity the PASI score cut off for considering mild, moderate and severe psoriasis was very variable. Some Authors [22, 38] considered PASI < 3 or <7, between 3/7 and 10/12, and >10/12 as mild, moderate and severe psoriasis, while other Authors [32, 42, 45] used the cut off of PASI < 20 or <25, between 20/25 and 49, and >50 as mild, moderate and severe psoriasis.

Again, short duration of follow-up and study design could affect the results of the intervention studies. In particular, for the parallel design studies (treated versus untreated patients or different treatments), the small sample sizes could have led to ineffective randomization and potential confounding, while in longitudinal (non controlled) trials it is difficult to draw any firm conclusion.

Finally, although investigators often excluded individuals with any other systemic diseases (diabetes mellitus, abnormal lipid profile, cardiovascular, kidney and/or liver diseases) [18–24, 28–32, 36–40, 61], the wash-out period (treatment free period before blood collection) was very variable (2 weeks - 6 months) [20, 21, 23, 27, 29–32, 36–40, 44, 59, 61]. Furthermore, only few studies specified that subjects did not receive lipid-lowering drugs [31] or antioxidant and vitamin supplements [20, 31, 37, 39, 40, 59]. In particular, Ikonomidis et al. [35] reported that all patients with psoriasis received treatment with cyclosporine 2.5-3 mg/kg daily. In addition, some subjects had hypertension, dyslipidemia and were treated with β-blockers, angiotensin-converting enzyme inhibitors, calcium channel blockers, diuretics and statins [35].

### Methodological consideration on peroxidation biomarkers and total antioxidant capacity

All the reviewed studies used old markers, such as peroxides, dienes, TBARS or MDA, that are progressively being replaced by new one isoprostanes [71] and all studies measured TAC by using a single assay, which imposes the chemical limitations of that assay [72, 73].

Antioxidant activity can be monitored by a variety of assays with different mechanisms, including hydrogen atom transfer (HAT), single electron transfer (SET) and reducing power, among others [74, 75]. Oxygen radical antioxidant capacity (ORAC) is the most common HAT method and other similar HAT-based methods sharing the same principle and common features of ORAC assay include total radical trapping antioxidant parameter (TRAP) and crocin bleaching assays [74].

In these methods the peroxyl radical produced by a generator reacts with a probe resulting in the loss of fluorescence or absorbance, that are recorded by decay curves [74]. Commonly used peroxyl radical generators include a group of azo compounds such as the hydrophilic 2,2′-azobis(2-amidinopropane) dihydrochloride (AAPH) and the lipophilic 2,2′-azobis(2,4-dimethylnaleronitrile (AMVN). A standard antioxidant, usually Trolox (a vitamin E analogue), is used as reference and ORAC values of the tested antioxidants are reported as Trolox equivalents [74]. Also SET-methods typically used Trolox as standard antioxidant [74]. The Trolox equivalent antioxidant capacity (TEAC) assay is the most used method in the reviewed studies and is classified as SET-based method [74]. The assay measures the ability of antioxidants to scavenge the stable radical cation 2,2′-azinobis(3-ethylbenzothiazoline-6-sulphonic acid (ABTS), a blue-green chromophore with maximum absorption at 734 nm which decreases in its intensity in the presence of antioxidants [74]. Similarly 2,2-Diphenyl-1-picrylhydrazyl (DPPH) is a stable chromogen radical with a deep purple colour [74].

The reducing power of antioxidants is measured through redox reaction with various metal ions, such as iron (ferric reducing antioxidant potential, FRAP), and copper (Cupric reducing antioxidant capacity, CUPRAC) [74]. Also the methods of Erel et al. for the measure of TAS and TOS/TOC involve oxido-reduction of iron [76, 77]. The TOS/TOC assay is based on the oxidation of ferrous ion to ferric ion in the presence of various oxidant species in acidic medium and the measurement of the coloured complex ferric ion-xylenol orange [76]. The TAS/TAR assay is based on the production of hydroxyl radical via Fenton reaction, and the rate of the reactions was monitored by following the absorbance of coloured dianisidyl radicals. The mixture of ortho-dianisidine, ferrous ammonium sulfate and hydrogen peroxide solution produced oxidized o-dianisidine molecules into dianisidyl radicals, leading to a bright yellow-brown colour development and antioxidants suppressed the colour formation [77]. The TOS/TOC assay is calibrated with hydrogen peroxide and results are expressed in terms of micromolar of hydrogen peroxide equivalent per litre (μmol H2O2 Eq/l) [76], whereas the TAS assay is calibrated with a stable antioxidant standard solution, which is traditionally the Trolox, and results were expressed as mmol Trolox Eq/l [77].

Although different TAC assays sometimes did not correlate with each other [73], even considering the same method or methods with similar mechanisms, the results were conflicting.

Both decreased [34, 37, 53, 56] and unchanged [54, 55] TAC levels were reported by using TAS method. When TAC was evaluated by TEAC neither significant correlations with PASI scores [30] nor differences between active and inactive psoriasis were reported [48, 49]. Although severity wise decrease in TAC levels, by using crocin bleaching [32], both increased [62] and unchanged [21] ORAC values were reported after anti-TNF-α treatment. Severity wise decrease in TAC levels, measured by FRAP [36] but not by CUPRAC [22].

TAC methods are strongly influenced by the presence of products of catabolism, such as bilirubin and uric acid [78] (Fig. 1). Although the role of uric acid and bilirubin in the prognosis of oxidative stress-related pathologies is still controversial [79, 80], hyperuricemia affected 20 % of patients with psoriasis [81] and high levels of bilirubin were found in some of the reviewed cases, probably due to red blood cells’ damage [27, 47]. Either decreased [47] or unchanged [27] TAC values in patients with psoriasis as compared with healthy subjects were reported. In addition, Severin et al. [50] found increased levels of both bilirubin and urate and TAC values that did not differ from controls. High level of bilirubin could not only underestimate oxidative stress assessed with TAC methods, but also overestimate oxidative stress by using TBARS assay (Fig. 1). In fact, it is known that bilirubin, as well as other compounds like sugars or amino acids, is able to react with thiobarbituric acid [75]. From that, the interference of bilirubin and uric acid must be taken into account both in the interpretation of the results and in the evaluation of the clinical significance of TAC and TBARS (Fig. 1).

On the other hand, increased levels of TOS were reported [30, 37, 53].

Regarding the relationship between cellular and plasma TAC and markers of lipoperoxidation, Barygina et al. [21] found higher levels of TBARS and lower levels of ORAC in both plasma and cell lysates of white blood cells. Besides, the increase of TBARS and the decrease of ORAC, were accompanied by an increase of protein carbonyl content (marker of oxidative protein damage) in plasma and by an increase of the redox glutathione ratio (GSSG/GSH) and of the cellular lipoperoxidation estimated using the fluorescent dye 4,4-difluoro-5-(4-phenyl-1,3-butadienyl)-4-bora-3a,4a-diaza-s-indacene-3-undecanoic acid (BODIPY 581/591 C11) [21]. In addition, the NOX activity and the intracellular ROS, measured with the fluorescent dye dihydrochlorofluorescein diacetate (H2DCF-DA), were also higher in psoriatic patients [21]. Furthermore, all these markers were improved after anti-TNF-α treatment. Therefore, although the limitations of TAC and plasma peroxidation markers, they reflect the cellular radical damage induced by oxidative burst.

### Relationship between cellular and plasma oxidative stress: metal redox status affects antioxidant enzymes

Antioxidant enzymes have a significant impact on the redox status, which is not reflected in the assays of isolated plasma [73]. In fact, antioxidant defenses of the body are composed of molecular and enzymatic players; however, the composition of the network markedly differs in terms of concentration and components in different environments [8]. Protection at the cellular level is mainly guaranteed by enzymes, whereas in plasma, non-enzymatic antioxidants (including low-molecular weight compounds such as uric acid, bilirubin, thiols, vitamin E, ascorbic acid, and carotenoids) play the major role [8]. The latter were measured by TAC methods, but their relationship with cellular antioxidants is often a neglected aspect.

Superoxide dismutase (SOD) catalyzes the one-electron dismutation of superoxide into hydrogen peroxide and oxygen; catalase (CAT) then operates the two electron-dismutation into oxygen and water [8, 16] (Fig. 1). Glutathione peroxidase (GPX) is involved not only in hydrogen peroxide removal but also in converting lipid hydroperoxides into their corresponding alcohols [8].

In the reviewed studies, data on antioxidant enzymes are controversial. Although CAT or SOD were decreased in the majority of the studies [20, 28, 29, 32, 36, 40–42, 45, 56, 58], these enzymes were unchanged in one study [47] and increases were reported in CAT [40, 41] or SOD [23, 33]. Gavan et al. [33] found increased levels of SOD in patients with psoriasis, which were significantly decreased after glucocorticosteroid application, despite the increase of TAC. Although superoxide production was higher in both patients with severe erythrodermic (EPS) and arthropathic (PSA) forms of psoriasis, increased SOD and CAT levels were found in EPS, and decreased SOD levels were found in arthropathic PSA forms of psoriasis [82], suggesting that only severe forms of psoriasis are associated with decreased enzymatic antioxidant defences. In fact it has been reported that the blood SOD level decreased significantly with increasing severity of psoriasis [20].

In addition it has been found that the activity of blood CAT increased, and activity of blood SOD decreased with increasing severity of psoriasis [40].

On the other hand, although decreases of GPX have been reported in psoriasis [45, 56–58], it has been reported that only the group of patients who did not respond to the treatment with efalizumab selectively showed a peculiar up-regulation of polymorphonuclear cells (PMN)-associated GPX after 12-week, both when compared to GPX levels of the same group of patients before treatment and also when compared to GPX levels of the groups of responders at the same time-point [44]. In addition, CAT activity in PMN was significantly lower in this group of patients after 12-week when compared to responders at the same time-point, whereas SOD remained unchanged [44]. When examined in erythrocytes, the high baseline GPX was found significantly reduced exclusively in the responding patients following a 12-week therapy, but no relevant changes were found in the other antioxidant enzymes [44]. Therefore, Pastore et al. [44] suggested that high hydroperoxide levels are involved in psoriasis persistence. Also Matoshvili et al. [41] suggested that the statistically significant increase in the activity of CAT in psoriatic patients reflects a high concentration of peroxides.

In this context it is known that SOD, CAT and GPX are regulated at transcriptional levels by cellular redox status, through a mechanism that involves the interaction between the antioxidant responsive elements (ARE) and the nuclear factor-erythroid 2-related factor 2 (Nfr2) [83, 84] (Fig. 1). Under physiological conditions, Nfr2 is bound to kelch-like ECH-associated protein-1 (Keap1) and thereby sequestered in the cytoplasm, whereas under conditions of oxidative stress, Nfr2 dissociates from Keap1, translocates to the nucleus and induces the transcription of antioxidant enzymes. The cysteine residues on Keap1, which are ultrasensitive to electrophiles, are critically important for the binding with Nrf2 [83].

Also nuclear factor- kappa B (NF-kB) is activated by ROS, through the degradation of its inhibitor IkB, and then migrates to the nucleus stimulating the expression of its target genes, such as inflammatory cytokines [84] (Fig. 1). The common mechanisms of activation and the interplay between Nfr2 and NF-kB have been reviewed and it has been suggested a potential concerted modulation of antioxidant and inflammatory pathways via upstream mitogen-activated protein kinases (MAPK) [84]. MAPK [85] and NK-kB [86] have a pathogenic role in psoriasis.

It is well known that iron and copper could induce both NF-kB and Nfr2 pathways, through production of ROS [87–94] (Fig. 1). Increased levels of copper [95, 96] and free reactive iron [34] were found in psoriatic patients. When comparison of copper data was done between groups of psoriasis severity, a significant difference was found between mild, moderate and severe groups [96].

Within TAC methods, FRAP and CUPRAC matches the antioxidant capacity to the reducing ability [74, 75] and also the methods of Erel for the measure of TAS and TOS/TOC are based on the iron redox status [76, 77] (Fig. 1). Although reducing and antioxidant capacity are related, it must be taken into account that hydroxyl radical, the initiator of the lipid peroxidation, is produced from the reaction between reduced iron or copper and hydrogen peroxide [75] (Fig. 1).

Therefore, a decrease in the metal reducing power could be more likely beneficial than detrimental in conditions of high levels of copper or iron and low levels of antioxidant enzymes (such as psoriasis).

## Conclusion

Psoriasis is associated with systemic oxidative stress. Overall the reviewed case–control (Table 1) and intervention (Table 2) studies suggest that peroxidation markers are more sensitive than TAC in the evaluation of oxidative stress in psoriasis (Fig. 1). Furthermore, it seems that biological drugs could be the better choice in the treatment of psoriasis (Fig. 1). However, in this review we pointed out the limitations of TAC and plasma peroxidation markers and the low clinical significance in cases of high levels of bilirubin, uric acid, copper or iron and low levels of antioxidant enzymes (Fig. 1).

Concerning other possible markers of oxidative stress, the ischaemia-modified albumin (IMA), as detected using the albumin cobalt-binding test, was registered by the United States Food and Drug Administration as a marker of myocardial ischemia and has been evaluated in two cross-sectional studies in psoriasis [97, 98]. IMA levels were higher in patients with psoriasis than in healthy subjects [97, 98] and showed a significant positive correlation with PASI score [97]. However, it has been pointed out that further studies for normal population distributions by gender and ethnicity, and an optimum cut-off value are still required for IMA [99].

In conclusion our review suggests that new methods should be developed in order to evaluate systemic oxidative stress in psoriasis.

## Abbreviations

8-OHdG, 8-hydroxy-2’-deoxyguanosine; AAPH, 2,2′-azobis(2-amidinopropane) dihydrochloride; ABTS, 2,2′-azinobis(3-ethylbenzothiazoline-6-sulphonic acid; ALT, alanine aminotransferase; AMVN, 2,2′-azobis(2,4-dimethylnaleronitrile; AOP, antioxidant potential; ARE, antioxidant responsive elements; BB UVB, broad-band UVB; BODIPY 581/591 C11, 4,4-difluoro-5-(4-phenyl-1,3-butadienyl)-4-bora-3a,4a-diaza-s-indacene-3-undecanoic acid; CAT, catalase; CHOL, cholesterol; CRP, C reactive protein; CUPRAC, cupric reducing antioxidant capacity; CVD, cardiovascular diseases; DPPH, 2,2-Diphenyl-1-picrylhydrazyl; EPS, erythrodermic psoriasis; ESR, erythrocyte sedimentation rate; Fe, iron; FRAP, ferric reducing antioxidant potential; GPX, glutathione peroxidase; GSSG/GSH, redox glutathione ratio; H2DCF-DA, dihydrochlorofluorescein diacetate; H_2_O_2_, hydrogen peroxide; HAT, hydrogen atom transfer; HDL, high-density lipoprotein; IFN, interferon; IkB, inhibitor of nuclear factor- kappa B; IL, interleukin; IMA, ischaemia-modified albumin; iNOS, inducible nitric oxide synthase; Keap1, kelch-like ECH-associated protein-1; LDL, low density lipoproteins; MAPK, mitogen-activated protein kinases; MDA, malondialdehyde; MMF, mycophenolate mofetil; MPO, myeloperoxidase; MS, metabolic syndrome; MTX, methotrexate; NB UVB, narrow-band UVB; NEAC, non-enzymatic antioxidant capacity; NF-kB, nuclear factor- kappa B; Nfr2, nuclear factor-erythroid 2-related factor 2; NOX, NADPH-oxidase; O^2^-•, superoxide radical; OH•, hydroxyl radical; ORAC, oxygen radical antioxidant capacity; ox-LDL, oxidized low density lipoproteins; PASI, Psoriasis Area Severity Index; PMN, polymorphonuclear cells; PSA, arthropathic psoriasis; PUVA, UVA with psoralen; ROO•, peroxyl radicals; ROS, reactive oxygen species; SET, single electron transfer; SOD, superoxide dismutase; TAC, total antioxidant capacity; TAR, total antioxidant response; TAS, total antioxidant status; TBARS, thiobarbituric acid reactive substance; TEAC, trolox equivalent antioxidant capacity; TG, triglycerides; Th, T-helper; TNF, tumor necrosis factor; TOC, total oxidant capacity; TOS, total oxidant status; TRAP, total radical trapping antioxidant parameter; UV, ultraviolet; VAP-1, vascular adhesion protein-1
